# Obstetric fistula in Southern Sudan: situational analysis and Key Informant Method to estimate prevalence

**DOI:** 10.1186/1471-2393-13-64

**Published:** 2013-03-12

**Authors:** Alma J Adler, Samantha Fox, Oona M R Campbell, Hannah Kuper

**Affiliations:** 1 March Centre for Maternal, Reproductive and Child Health, London School of Hygiene & Tropical Medicine, London, UK; 2 International Centre for Evidence in Disability, Clinical Research Department, London School of Hygiene & Tropical Medicine, London, UK

## Abstract

**Background:**

Obstetric fistula is a severe condition which can have devastating consequences for a woman’s life. Despite a considerable literature, very little is known about its prevalence. This project was conducted to carry out a situational analysis of fistula services in South Sudan and to pilot test the Key Informant Method (KIM) to estimate the prevalence of fistula in a region of South Sudan.

**Methods:**

Key stakeholder interviews, document reviews and fistula surgery record reviews were undertaken. A KIM survey was conducted in a district of Western Bahr-el-Ghazal in January 2012. One hundred sixty-six community-based distributors, traditional birth attendants and village midwives were trained as key informants to identify women with fistula in the community. Women identified were subsequently examined by an obstetrician and nurse to verify whether they had a fistula.

**Results:**

There were limited fistula repair services in South Sudan. Approximately 50–80 women per year attend periodic campaigns, with around half having a fistula and receiving a repair. On average a further 5 women a year received fistula repair from hospital services. Ten women with potential fistula were identified via KIM; all confirmed by the obstetrician. Of these, three were from the survey area, which had 8,865 women of reproductive age (15–49 years). This gives a minimal estimated prevalence of at least 30 fistulas per 100,000 women of reproductive age (95% CI 10–100).

**Conclusions:**

Routine fistula repair services available do not meet the population’s needs. The pilot study suggests that KIM can be used to identify women with fistula in the community. Data on fistula are generally poor; the KIM methodology we used in South Sudan yielded a lower fistula prevalence than estimates reported previously in the region.

## Background

Obstetric fistula (fistula from here on) is an “abnormal opening between a woman’s vagina and bladder and/or rectum, through which her urine and/or faeces continually leak” [1]. Fistulae are generally caused by long obstructed labours. Such labours can last for days and in most cases the baby dies [2,3]. During an obstructed labour, the baby’s head becomes lodged in the pelvis and the pressure from the head can cut off the blood flow to the surrounding tissues causing them to necrotize and form a hole. Surgical repair of fistula is possible even if the fistula has been present for some time [1]. Closure rates of 85-95% for those operated on have been reported in a number of case series [4]. However, it is unclear how many women are considered “inoperable” before an operation is attempted.

The potential consequences for women who suffer from fistula are social, emotional and physical [3]. The woman may have sores on her genitals due to urine dermatitis, be unable to have sex, and stop or have irregular periods [4]. These factors, and the associated smell, may lead to social problems, loss of ability to work and estrangement from spouses, family and society [4]. There is also limited evidence of an increased risk of depression [5]. The time and resources required by women to keep clean has also been found to have a major impact on women’s lives [6].

Fistulae are thought to be relatively rare globally, with most cases originating in low income countries in women lacking access to intrapartum care [2]. The WHO estimates two million women have fistula globally [1], but do not state their reliable sources. There are few reliable prevalence estimates because of fistula’s rarity and the remoteness of the areas where sufferers tend to live. Most studies stating a prevalence of fistula are based on self reports, personal communications with surgeons, studies from advocacy groups or reviews of hospital services without denominators [7]. A commonly cited statistic, that fistula occurs in 200–500 per 100,000 deliveries, originates from a personal communication with a fistula surgeon working in East Africa [8]. Incidence in the MOMA study [9] of eight centres in six countries in west Africa is 10 per 100,000 pregnancies overall (denominator 19,342 pregnancies) and 120 in rural areas (denominator 1,543 pregnancies). Community-based prevalence estimates from Gambia [10] are 96 per 100,000 women of reproductive age (denominator 1038 women) and from Ethiopia [11] (treated and untreated) are 203 per 100,000 women of reproductive age (denominator 27,090 women). We did not find estimates for South Sudan or for Sudan before separation.

Because fistulae are rare, conducting a household survey to estimate prevalence precisely would involve an extremely large sample size and would require high specificity of questions or approaches to ascertaining fistula. The Key Informant Method (KIM) offers an alternative method for estimating the prevalence of rare conditions, including childhood blindness and childhood disability [12,13]. Key informants are trained across a defined geographic area to identify all potential cases of a condition. These cases are then examined by a clinician to verify case status. The prevalence of the condition is estimated as the number of cases divided by the population at risk in the area.

Following 50 years of war, South Sudan became independent in June 2011. Its population is 8.26 million [14] and is one of the world’s least developed countries. A recent report found 33 functioning hospitals in the whole country, with only 16% of all health facilities having electricity [15]. It is estimated that there is only one doctor per 100,000 people in South Sudan; substantially lower than the 20 per 100,000 recommended by WHO [16]. South Sudan reports some of the worst health statistics in the world, with an Infant Mortality Rate of 102 per 1000 live births and a Maternal Mortality Ratio of 2054 per 100,000 live births [14]. Only 19% of women are estimated to give birth with a skilled birth attendant, 12% to give birth in health facilities, and 13% to use antenatal care [17]. For these reasons, South Sudanese women are considered to be at high risk for obstetric morbidities, including fistula.

This work was conducted with three aims: to understand the existing availability of fistula services in South Sudan, to assess whether KIM is an effective method for finding women with fistula, and to estimate the prevalence of fistula in women of reproductive age in a region of South Sudan.

## Methods

### Situational analysis

**Figure 1:**
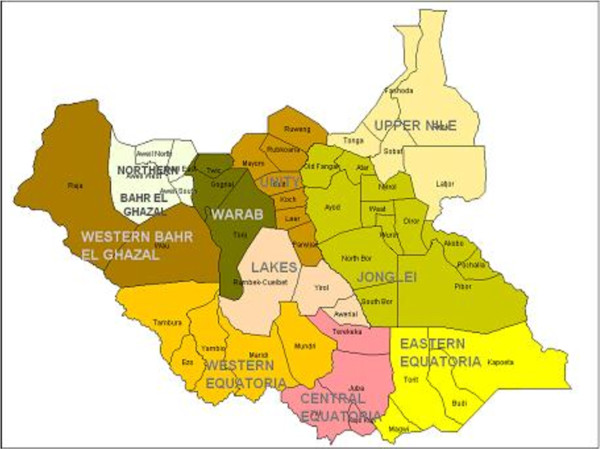
Map of South Sudan.

We undertook a situational analysis of fistula services in South Sudan. First, we interviewed stakeholders to identify health facilities in South Sudan where fistula surgeries were undertaken routinely or where campaigns had been carried out. We reviewed records of two of the three teaching hospitals in South Sudan (Juba and Wau Figure 1), and of all the fistula campaigns conducted between 2006 and 2011. We did not visit the third teaching hospital in Malakal for security reasons, but gathered information from Malakal, other hospitals where operations were thought to have been performed and non-governmental organisations (NGOs) through telephone and email communication. We extracted data using a standardised data collection tool to estimate the annual numbers of fistula surgeries conducted in South Sudan and assess patient characteristics, details of fistula and risk factors.

We also conducted semi-structured interviews with key stakeholders in the government, hospitals and NGOs about fistula repair services in South Sudan. These interviews were generally conducted in English, with translators used when necessary.

### Key Informant Method

#### Setting

We conducted a KIM study in January – February 2012 to estimate the prevalence of fistula in the area around Wau, the second largest city in South Sudan, and the capital of Western Bahr-el-Ghazal State (WBG). This area has an estimated population of 150,000 and was chosen as it had established networks of people who could act as key informants and is relatively secure. In addition, it is the location of one of the three teaching hospitals in the country and has two nurses and a doctor who had received fistula repair training so that surgery could be provided to women identified with fistula.

#### Recruitment of key informants

Two pre-existing networks of women were used to recruit key informants. The first was Population Services International’s (PSI) network of 94 community-based distributors (CBDs) in two Payams of WBG (Bazia and Bagari) covering a total population of about 25,300 people. The CBDs had undergone a two-week training to enable them to diagnose malaria and deliver medication to their community. The second was the Ministry of Health’s (MoH) network comprising village midwives and trained traditional birth attendants (TBAs). They had received 18 and three months training respectively and knew the communities in question. We requested that village midwives and TBAs from one Payam (Besselia) and two Bomas (Darajat East and West), representing 19,700 people attend training.

#### Training of key informants

Five half-day training sessions were held over three days by a nurse from Wau teaching hospital who had attended the Fistula Hospital training in Addis Ababa and a MoH midwife, with support from one of the authors (AJA). The training covered the causes and consequences of fistula, how to recognise a woman who may have fistula and how to discuss the issue sensitively and confidentially.

#### Identification of women with fistula

After training, key informants returned to their community to identify potential cases with fistula among women aged 15–49, through their own knowledge and by talking to women and asking them whether they or other women of their acquaintance experience symptoms of fistula (including excessive washing of clothes, urine leakage or smell). They informed their local coordinator of any suspected cases, who then passed the information onto a project coordinator at the Wau MoH.

Women who were eligible cases in this study had to be resident in one of these Payams or Bomas, had to have had at least one pregnancy, and be of reproductive age (defined in this study as between the ages of 15–49).

#### Examination of potential cases of fistula by obstetrician

Potential cases of fistula were verified in a mobile clinic with appropriate levels of hygiene, fully equipped to detect fistula, and staffed by an obstetrician and the trained nurse who had assisted with the training. Key informants told the potential cases of fistula when the mobile clinic would be at their local health clinic.

The women were screened using a speculum and the dye test, (inserting dye into the bladder to see if it comes out of the vagina) [1]. At the time of their screening, the women were interviewed by the doctor and the nurse using a standard questionnaire to collect data on literacy, parity, current age, age at onset and impact of fistula on their lives.

## Data analysis

### Situation analysis

The data collected from the record and campaign reviews were collated into an Excel spreadsheet and analysed using Stata. The interviews with stakeholders were analysed using the framework approach, a method widely used by health and social researchers to gather policy and practice orientated findings [18].

### KIM

The numbers of women of reproductive age for WBG and South Sudan as a whole were taken directly from the South Sudan Household Survey [17]. The number of women of reproductive age in the sampled districts was estimated using the total number of individuals resident, multiplied by 19.7% which was the percentage of women of reproductive age in WBG reported by the survey. The number of women identified from the study areas with fistula was divided by the women estimated to be of reproductive age, then multiplied by 100,000 to obtain a prevalence per 100,000 women of reproductive age. We used this prevalence to estimate the number of women with fistula in WBG and in the country. We conducted sensitivity analysis by varying the percentage of the population women of reproductive age from 19% to 22%.

### Ethical approval and treatment

Ethical approval for the study was gained from the London School of Hygiene & Tropical Medicine and the Ministry of Health of the Republic of South Sudan. All women identified by key informants were provided with reusable incontinence pads. Informed consent to participate in this study was obtained from all participants by a Hamlin trained nurse. If the women were unable to read, they were read the form and gave their consent. All women identified with either fistula or other conditions amenable to treatment were referred for surgical correction funded by the UNFPA.

## Results

### Situational analysis

The situational analysis highlighted that South Sudan has limited fistula repair services. Most fistula services in South Sudan consist of semi-regular campaigns where an expatriate team is commissioned to provide surgical repair for a limited time at a location in South Sudan. The service is advertised through media campaigns, and hospitals are asked to refer women who present with fistula to the campaign. We identified eight campaigns between 2006 and 2011: five in Juba, one in Rumbek, and two in Mapuordit (Table 1). Five were run by the UNFPA, two by AMREF, and one where we were unable to find any further information. The campaigns did not have detailed records, and where headline figures were available, they did not always correlate with the operation records kept by the hospital where the campaigns had taken place. Greater details were available for campaigns in 2009 and 2010, the details for 2010 are presented in Table 2. The number of women with fistula operated on recorded in the detailed records for 2009 and 2010 were substantially lower than the “total operations” quoted in UNFPA reports, as these included operations carried out for other conditions, notably fourth degree perineal tears and “other” conditions such as prolapse. Reports from earlier campaigns may overestimate the numbers of fistula repaired for similar reasons. According to available information, approximately 150 women received a repair between 2006 and 2012 (Table 1), however, this estimate is uncertain as there may have been women included in this figure who received surgery for a condition other than fistula and women who received a fistula repair in a campaign which were we were not aware of. A further 120 women were on “waiting lists” for treatment held by hospitals and NGOs, but this is likely to be an incomplete list.

**Table 1:** Details of fistula repair surgery carried out in South Sudan, 2006-2011

**Year**	**Organiser**	**Location of campaign**	**Repair team**	**Advertising**	**Composition of the campaign**	**Number of women attending**	**Number of fistula operated UNFPA record (operating book record**	**Number of fistula successfully operated on**
2006	UNFPA /MOH	Juba	Nigeria	Media campaign	Counselling	Not reported	19 (16)	Not reported
					Repair			
					Life skills			
2007	UNFPA/ MOH	Juba	Nigeria	Media campaign	Counselling	57 from 6 states	33 (28)	29
					Repair			
					Life skills			
2008	UNFPA/ MOH	Juba	Nigeria	Media campaign	Counselling	40	(6)	Not reported
					Repair			
					Life skills			
2009	UNFPA/ MOH	Juba	Nigeria	Media campaign	Repair	28	19 (44)	Not reported
2009	UNFPA /MOH	Juba	St Mary’s Hospital, Isle of Wight, UK, Juba Link	Not reported	Repair	Not reported	15 (the records may be included in the figure above)	Not reported
2010	AMREF/ WAHA	Mapuordit	AMREF/ WAHA/ MSF	Not reported	repair	14	Not reported	Not reported
2010	UNFPA/ MOH	Rumbek	Hamlin Fistula Hospital Ethiopia and AMREF Fistula Surgeons	Media campaign. Governor of state launched.March	Repair	81(39 with fistula)	32	28
2011	Operating book record	Juba	Not reported	Not reported	Repair	Not reported	11	Not reported
2011	AMREF	Mapuordit	AMREF	Not reported	Repair	12	Not reported	Not reported

**Table 2:** Details of 2010 UNFPA campaign conducted in Rumbek

**Diagnosis**	**Number of diagnosis (%)**	**Operated (%)**	**Success (%)**
Vesicovaginal	28 (35)	22 (79)	20(91)
Rectovaginal	4 (5)	4 (100)	4 (100)
Recto and vesicovaginal	7 (9)	5 (71)	4 (80)
**All fistula**	39 (48)	32 (82)	28 (88)
4th degree perineal tear	21 (26)	21 (100)	21 (100)
Other operable conditions	21 (26)	14 (67)	10 (71)
Total	81	66(81)	59 (89)

Approximately 20 health professionals have been trained in simple fistula repair, but only one doctor has recently started conducting fistula repairs (seven repairs in the previous year). There were challenges for those trained to provide services because of the lack of appropriate equipment, facilities and ongoing educational support.

All the stakeholders working in the government and health system interviewed felt that fistula was a big problem in South Sudan, despite relatively few cases presenting to facilities. All clinical staff and policy makers wished to have permanent fistula repair services in South Sudan. They stated that although campaigns had enabled some access, their short-term nature and limited reach was an issue. They stated that only women with direct or indirect access to radio messages, or who had the social or financial resources to travel to the hospital, were likely to have been able to access care. Substantial barriers to providing a service within South Sudan were described; including lack of skills and facilities, and lack of awareness of fistula’s amenity to treatment in the community. Where clinicians had received training or had gained experience during the campaigns, they had in the main not used these skills subsequently. This was thought to result from a number of factors including commitment by the individual, organisations not utilising the skills, or people changing jobs. Clinicians and policy makers identified two main ways to tackle fistula, by improving obstetric services to prevent and raise awareness of fistula and by developing permanent fistula repair services. At the community level by contrast, the key informants had very little knowledge of the condition from their communities, with only two cases of fistula being remembered by key informants.

### Key Informant Method

#### Results from training

We expected 94 key informants from Bazia and Bagari Payams to attend the training; 158 women and eight men attended. The attendants were CBDs, TBAs and midwives. We expected approximately 20 midwives from Bessila and Darajat East and West, however 51 midwives attended. These included women from outside our defined study area.

Ten women were identified as potential cases of fistula by the key informants. All were confirmed as having fistula after gynaecologic examination by the obstetrician, giving a positive predictive value of 100%. Of these, only three women came from the target population and were eligible to be included in the prevalence estimate. Three came from another state (Warrap) and four came from other areas in WBG.

Characteristics of the women with fistula are found in Table 3. All of the women reported a long obstructed labour prior to fistula formation. Half the women had genital sores. All women reported either less intercourse than before fistula formation or no intercourse at all. Women had been suffering from their fistula for between one month and 37 years; two had previously attempted repair. The median age at onset was 16 years with a range of 13–27 years. It is difficult to determine if the three women that were in our predefined area were representative of the larger sample because of the small sample size.

**Table 3:** Characteristics of women with fistula identified in KIM

**Characteristic**	**Total fistula sample**	**Eligible fistula cases**
	**N=10**	**N=3**
Mean age (years)	29	42
Median age (years)	21	48
Marital status		
Married	60%	67%
Divorced	10%	0%
Widowed	30%	33%
Median Height (cm)	153	155
Median Weight (kg)	50.2	51.7
Median BMI (kg/m^2^)	18.5	21.5
Literacy		
Can read	10%	0%
Can write	10%	0%
Can sign name	20%	33%
Illiterate	60%	67%
Median time since onset of fistula (range) (years)	**3.5 (1 month −37 years)**	21 (9 years −36 years)
Median age at onset of fistula (range) (years)	**16.2 (13–27)**	19 (14–27)
Type of fistula		
Vesicovaginal fistula	70%	67%
Rectovaginal	10%	0%
Rectovaginal and vesicovaginal	20%	33%
Parity		
1	70%	33%
2	0%	0%
>2	30%	67%
Previous attempted repair	20%	33%
Genital sores	50%	100%
Irregular menses	50%	33%
Less intercourse than before	30%	67%
No intercourse	70%	33%
Attended for verification	100%	100%

The population in the selected area was 45,000, resulting in an estimate of 8865 women of reproductive age. As we identified three cases of fistula, the estimated prevalence is at least 30 per 100,000 women (95% CI 10–100 per 100,000 women. This suggests that there are at least 619 women with untreated fistula in South Sudan. Because of the low prevalence, sensitivity analysis did not change our results. However, the prevalence of fistula is likely to vary across South Sudan, according to population and cultural characteristics and access to services.

## Discussion

We estimated a prevalence of fistula of at least 30 per 100,000 women of reproductive age in WBG, suggesting that if the results are extrapolated, there are at least 619 (128–1807) untreated women in South Sudan. However, the findings from our small sample in WBG are unlikely to be generalisable to the whole country given the wide range of access to maternity services from state to state. In 2006 in WBG, 13.8% of women had a skilled attendant present at their birth, compared to the 4.9% in Eastern Equatoria [19] and 12% in the whole country [14]. The risk of obstructed labour and consequently of fistula may vary between regions. Therefore, we cannot be confident that this is a true reflection of the minimal prevalence across the country.

The quality and capacity of current fistula repair services are inadequate to meet the needs of the population. This is within a context of limited health services in general and particularly for women. The campaign system has been a good short term measure, however it has not provided consistent or equitable care and is insufficient to cope with the backlog of cases, with nearly as many cases on waiting lists as have been treated in the last six years. Clinicians’ and policy makers within South Sudan described the key barriers to providing consistent hospital-based services, which would require investment in human resources and facilities to overcome. There are currently clinicians who have been trained to carry out basic fistula repairs and these skills could be better utilised and supported to provide a service for South Sudan. Any resources used for fistula service development have to be carefully weighed up against the greater numbers of people who are without the most basic of health services. This is particularly true in light of the relatively low prevalence estimates obtained in our study. Nonetheless there is a need to facilitate the access of these women with an unmet need for services; awareness of fistula could be raised among the population as part of the overall package of obstetric-related health messages.

Our estimated prevalence of 30 per 100,000 is considerably lower than previous population-based estimates [8,10,11]. Given the poor maternal health indicators in South Sudan, one would expect at least as high a prevalence as other regions of Sub-Saharan Africa. There are several potential explanations for our finding.

Our estimate could be correct, with others being incorrect. Many of the prevalence estimates reported in the literature are not based on epidemiological studies and as a result, are likely to be inaccurate; if publications are advocating to address the issue, they may well have overestimated the prevalence. Alternatively, our estimates may be correct but unrepresentative as it is possible that our selected area was too close to Wau, and therefore the communities that we visited would have had greater access to maternal services and therefore a decreased risk of fistula. However, the communities were one hour’s drive with good vehicles from the city, which would not be possible for most women in the communities, and the roads used were only completed in 2010. Finally, it is also possible that as South Sudan reports having the highest maternal mortality in the world and has high rates of all cause mortality, the women with obstructed labour did not survive long enough to present with fistula.

Alternatively, it is possible we underestimate the prevalence for a number of reasons. First, the KIM may not be an effective method for complete ascertainment of cases of fistula, and we did not find all the eligible cases. KIM has been validated for use for other rare conditions including childhood disability and childhood blindness [12,13], but while these are often stigmatizing conditions, it may be that mothers will bring children for assessment but not come themselves. Previous work on women with fistula has emphasised their isolation from family, friends and community [2,20,21]. This could mean that women were “hidden” and unable to be identified. Our key informants particularly the CBDs, were key members of their community and we feel it would be unlikely that in close communities women with fistula would be completely hidden. It is also possible that women with fistula were identified but refused to attend verification. Again, the training of the key informants was designed to ensure that women were supported, reassured about confidentiality and given information about treatment. The women with fistula who did come forward did not report stigma as a major issue.

Since women from outside the target area presented for verification, this suggested to us that messages were effectively communicated across a wide area and some women at least were willing to come forward. Additionally, subsequent research conducted in the same area by a team with a social anthropologist identified further women in areas outside of the one we studied, but no additional women were found in the region we studied.

Finally the prevalence could be influenced by inaccurately estimating the denominator, which was obtained from recent surveys conducted by the government. There is some debate about how accurate the household surveys were. However even if we overestimated the population by 10,000 people (23%), this would still have only resulted in an estimate of 40 per 100,000 women of reproductive age, which does not raise the prevalence by much.

This is the first time that KIM has been used to identify maternal morbidities, and we believe that it is a promising tool for the future. We have shown that it is possible to find women with fistula using a KIM. It has the advantage of being much faster and cheaper than household surveys, as well as benefiting from community involvement. However it has the limitation of having to rely on key informants of varying ability to find the women and the possibility of missing eligible cases. The next step is to use this method in further studies, in other regions of South Sudan and Sub-Saharan Africa. Complementary qualitative data that have been collected from the key informants and women with fistula will offer further insights to improve the key informant methodology. This methodology has the potential to become a practicable method for estimating the prevalence of women with fistula, which will be invaluable in planning future services and ensuring equitable access. Further use of this method in other regions of the world, possibly in contexts where other methods are used as well, would enable the production of comparable estimates, to identify whether the low prevalence found by this study in South Sudan is accurate or a result of methodological limitations. It has the added advantage of having a clinincal assessment which confirms the diagnosis. It is well understood that using women’s self-reported morbidity will over estimate the prevalence of rare conditions, unless the specificity of the questions is very, very high.

This study was a preliminary assessment of fistula in a region of South Sudan, identifying limited services and quantifying the potential unmet need. This provides important information for future policy and service development; to ensure that fistula is integrated with other services, existing skills are built on and women with fistula are proactively identified to receive services. Any service developed needs to be considered within the context of limited resources and that current demand is less than may have been expected.

## Conclusions

Obstetric fistula is a severe condition which can have devastating consequences for a woman’s life and is generally associated with poor obstetric services. Despite many publications, little is actually known about the overall prevalence of fistula as there are very few population-based studies looking at the prevalence that include a physical examination. Despite awareness at the government level, the routine fistula repair services available to the women of South Sudan do not meet the needs of the population. This is within a context of overall poor medical services particularly for women. The pilot study we conducted suggests that KIM can be used to identify women that have fistula in the community, however this is the first time this methodology has been used for determining the prevalence of obstetric fistula and more research should be conducted to show its usefulness in other settings. Within a context of poor reporting of fistula within Sub-Saharan Africa, our findings suggest that fistula may be less common in South Sudan than other regions of Sub-Saharan Africa.

## Competing interests

All authors declare that they have no competing interests.

## Authors’ contributions

AJA and SF, wrote the grant (including design), conducted the research, analysed the data, and wrote the first draft of the paper. OC and HK contributed to the design and conception of the study, provided feedback on results and interpretation of the data, and commented on drafts of the manuscript. All authors approved the final manuscript.

## Authors’ information

Joint first author: Samantha Fox.

## Pre-publication history

The pre-publication history for this paper can be accessed here:

http://www.biomedcentral.com/1471-2393/13/64/prepub
